# Preparation of Microneedle Array Mold Based on MEMS Lithography Technology

**DOI:** 10.3390/mi12010023

**Published:** 2020-12-28

**Authors:** Jie Wang, Huan Wang, Liyan Lai, Yigui Li

**Affiliations:** College of Science, Shanghai Institute of Technology, Shanghai 201418, China; wangjie20201010@163.com (J.W.); wanghuan@sit.edu.cn (H.W.); lylai@sit.edu.cn (L.L.)

**Keywords:** microneedle array mold, micro-electro-mechanical system (MEMS), Photolithography, Galvanogormung, Abformung (LIGA), engraving process, Ultraviolet (UV) tilting rotary lithography

## Abstract

As a transdermal drug delivery technology, microneedle array (MNA) has the characteristics of painless, minimally invasive, and precise dosage. This work discusses and compares the new MNA mold prepared by our group using MEMS technology. First, we introduced the planar pattern-to-cross-section technology (PCT) method using LIGA (Photolithography, Galvanogormung, Abformung) technology to obtain a three-dimensional structure similar to an X-ray mask pattern. On this basis, combined with polydimethylsiloxane (PDMS) transfer technology and electroplating process, metal MNA can be prepared. The second method is to use silicon wet etching combined with the SU-8 process to obtain a PDMS quadrangular pyramid MNA using PDMS transfer technology. Third method is to use the tilting rotary lithography process to obtain PDMS conical MNA on SU-8 photoresist through PDMS transfer technology. All three processes utilize parallel subtractive manufacturing methods, and the error range of reproducibility and accuracy is 2–11%. LIGA technology produces hollow MNA with an aspect ratio of up to 30, which is used for blood extraction and drug injection. The height of the MNA prepared by the engraving process is about 600 μm, which can achieve a sustained release effect together with a potential systemic delivery. The height of the MNA prepared by the ultraviolet exposure process is about 150 μm, which is used to stimulate the subcutaneous tissue.

## 1. Introduction

One method to produce microneedle arrays (MNA) is micro-electro-mechanical system (MEMS) technology [1,2]. MNA refer to needle-like structures with a diameter of several tens of micrometers and a length of more than one hundred micrometers and are generally fabricated by micro-nano manufacturing technology. According to the manufacturing process, it is divided into the in-plane MNA and out-plane MNA; according to the material, it is divided into silicon MNA, metal MNA, ceramic MNA, hydrogel-forming MNA, and polymer MNA; according to internal structure, it is divided into solid MNA and hollow MNA [3,4]. Since the first report of the application of MNA in transdermal drug delivery (TDD) in the 1990s, the advantages of MNAs have attracted much attention. Especially in recent years, the gradual improvement of MEMS technology has made the establishment of MNA drug delivery system rapid [5,6]. The way of administration of MNA is different from traditional oral administration and intravenous administration. The MNA form a tiny channel on the surface of the human skin, so that drug can reach the specified depth of the skin, and have the advantages of accurate administration, high efficiency, simple operation, and pain-free [7,8]. It is precisely because of the many superiorities of MNA in TDD that people are constantly exploring and researching the combination of MNA and therapeutics, such as a vaccine, insulin, and low molecular weight heparin. With the development of biomacromolecules and nanomedicines, the demand for transdermal delivery of MNA is also increasing, and MNA have broad prospects for development [9,10,11].

In recent years, some new MNA manufacturing methods have emerged, such as Ultraviolet (UV) tilting rotary lithography [12], wet etching [13], and other methods [14]. In 2016, Takahashi et al. changed the height and contour of the microneedle by changing the inclination angle and exposure dose of ultraviolet rays. In 2017, they used the principle of light refraction to control the height of the microneedle by changing the bottom angle of the prism above the mask [15]. In 2019, Huang et al. proposed a new type of wireless biopotential acquisition using a microneedle array, using the KOH-cut-KOH method to prepare a wearable patch, which realized multi-channel biopotential monitoring [16]. Ultraviolet exposure technology is the continuation of photolithography technology. The use of UV exposure mask combined with etching technology can produce microneedles with different shapes, angles and surface profiles. This technology can effectively improve the strength and toughness of MNA, so that they can perform different functions in different fields in consideration of different process conditions. In response to market demand, three MEMS-based microneedle preparation methods are proposed.

This article summarizes and compares three types of methods for manufacturing MNA. The first process is our optimization and improvement of the previous method [17], and the remaining two processes are our latest research results. The first category is based on LIGA(Photolithography, Galvanogormung, Abformung) processing technology which includes X-ray lithography, electroforming, and plastic molding with the advantages of high radiant energy, good parallelism of light, and consistent spectral continuity, etc. LIGA technology is widely used in the field of microfabrication, precisely because the processing objects of MNAs include metals, ceramics, polymer materials, etc. [18]. The three-dimensional structure similar to the X-ray mask pattern is finally fabricated on the polymethyl methacrylate (PMMA) photoresist after twice moving X-ray lithography processes and development technique by used plain-pattern to cross-section technique (PCT). On the basis of twice moving X-ray lithography, the PMMA hollow MNA can be fabricated by a single fixed alignment X-ray exposure process [19]. As long as different X-ray mask gold absorber patterns are designed, different three-dimensional structures can be obtained, such as out-plane MNA, and in-plane MNA. PMMA MNA with high aspect ratio, sharpness and various shapes are successfully prepared by PCT at one time. The second type is the fabrication method of the female MNA master mold, the tip part and the column part of the MNA are fabricated by silicon wet etching and UV-LIGA techniques, respectively [20]. The PDMS female mold is fabricated by the PDMS secondary transfer technology with the master mold. After that, the PDMS female master can be used to prepare MNA of different polymer materials. These MNA are approximately 325 μm high and the density reaches 300 needles per square centimeter. The engraving process could determine the height of the MNA by adjusting the thickness of the SU-8 photoresist. In the experiment, the height of the column part of the SU-8 MNA is about 250 μm. The third method is to obtain a conical female MNA master mold on the SU-8 photoresist by tilting rotary UV lithography and conical MNA of PDMS were produced through PDMS transfer technology [21]. This method uses SUEX Dry Film Resist (DFR) instead of the traditional SU-8 photoresist and uses the thermal lamination process to replace the original glue rejection technology, which can produce microneedle arrays with better IC compatibility at low cost. Finally achieve the goal of saving time, efficient and convenient. The MNA prepared by LIGA process combined with pumps and valves can be used in microfluidic systems. The hollow MNA prepared by this process can be used for blood extraction and drug injection. The microneedle prepared by the engraving process can be used for drug loading, which makes it play a great role in the field of transdermal drug delivery to achieve a sustained release effect. The MNA prepared by the ultraviolet lithography process is used to gently stimulate the subcutaneous tissue and promote tissue proliferation, which makes it suitable for medical cosmetology. We use PDMS to transcribe and replicate MNA. By comparing the sizes of the manufactured and replicated MNA, we used the measurement method to report the reproducibility and accuracy of different methods, and the error range was 2–11% in terms of microneedle height.

## 2. Materials and Methods

### 2.1. LIGA (Photolithography, Galvanogormung, Abformung) Processing Technology

**Figure 1:**
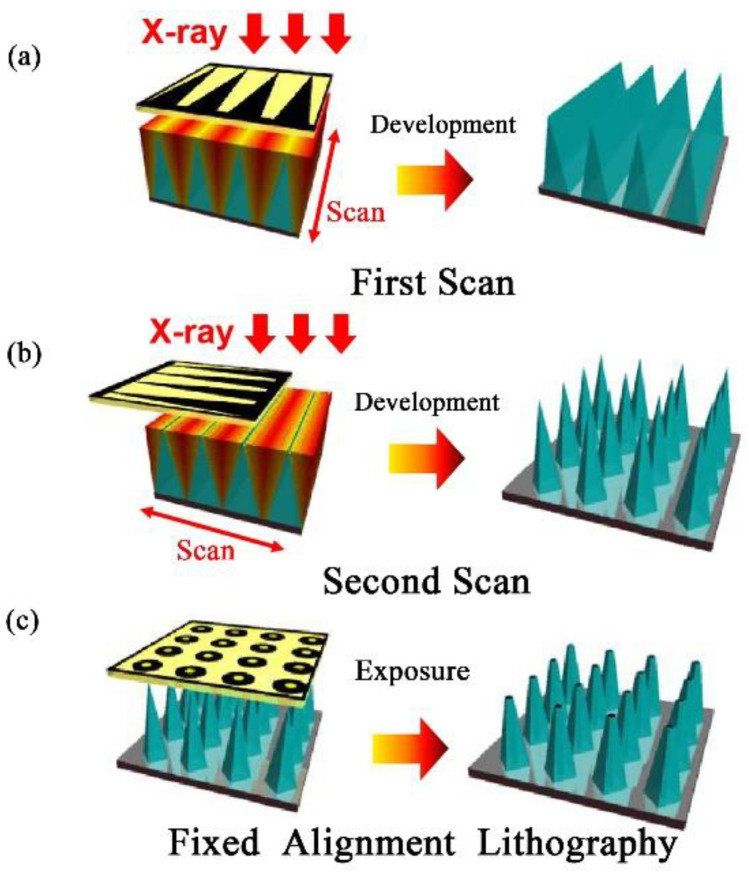
Schematic diagram of hollow polymethyl methacrylate (PMMA) microneedle arrays (MNA) prepared by Twice moving X-ray lithography process and fixed alignment X-ray exposure process. (**a**) First moving exposure. (**b**) Second moving exposure. (**c**) Fixed alignment lithography.

PCT technology is a method of manufacturing three-dimensional structures. The PMMA substrate can be moved repeatedly while X-ray scanning to form a three-dimensional structure, and the cross-section of the three-dimensional structure is similar to the pattern on the X-ray mask. The PMMA MNA was fabricated by synchrotron radiation lithography process, which was performed at the synchronous radiation source AURORA of the SR Center of Ritsumeikan University, Japan. The wavelength of SR light is between 0.15 and 0.73 nm, and the peak wavelength is 0.4 nm. The electron energy and maximum storage current of AURORA in the experiment were 575 MeV and 300 mA, respectively, the beam size was 30 × 5 mm, and the beam length was 3 m. The manufacturing schematic of the PMMA solid MNA is given in Figure 1. The first moving exposure is to obtain the PMMA triangular microprism structure, which is related to the shape of the absorber of the X-ray lithography mask. Because the X-ray absorption distribution of PMMA depends on the absorber pattern on the X-ray mask, a PMMA structure similar to the X-ray mask pattern can be formed. X-ray absorption in PMMA distribution depends on the absorber pattern on the X-ray mask, so the cross-section in the PMMA forms a structure similar to the X-ray mask pattern. After the first synchrotron radiation lithography, the second moving exposure is performed, and then the PMMA is rotated 90°, and the lithography is performed under the same lithography conditions. A high-precision mobile exposure table is used to complete the movement of PMMA photoresist, which includes a mask holder, a substrate holder and a driving table. The computer is used to control the driving table to make X, Y, Z three-axis precision movement. The range of movement in the X, Y, and Z directions is 50 mm, and the resolution is 50 nm; the resolution of the rotation angle in the mask plane is 0.0025°; the distance between the mask and the substrate is 20–100 μm. The manufacturing process of the hollow microneedle array is shown in Figure 1. On the basis of the two-step moving exposure process of Figure 1a,b, fixed alignment photolithography of the PMMA solid MNA to prepare the hollow MNA is shown in Figure 1c. In the manufacturing process of the PMMA MNA, the selected exposure dose for one directional scan was about 0.0045 Ah, after which the exposed PMMA structure appeared after 3 h of development with GG developer at 37 °C. The composition of the GG developer is 60% 2-(2-butoxy-ethoxy) ethanol (glycol ether), 20% tetra-hydro-1, 4-oxazine (hexa-atom ring compound) and 5% 2-amino-ethanol-1 (aminoethanol).

The shape of the PMMA MNA depends on lithography method and absorber pattern of the X-ray mask which has made of gold and polyimide layers with thicknesses of 3.5 μm and 50 μm, respectively. The gold material absorbs X-rays while the polyimide support film through X-rays. Therefore, MNA with different shapes and strengths can be fabricated by designing different X-ray mask absorber patterns and using different photolithography methods.

On the basis of the PCT, the out-planar metal MNA can be obtained by electroplating technology. Firstly, a PDMS solid MNA was fabricated by two PDMS transfer processes using a PMMA solid MNA as a master mold. Finally, a nickel metal MNA was fabricated by performing a nickel-plating process using the PDMS solid MNA.

Prior to electroplating the metallic nickel, a layer of chromium copper seed with a thickness of 250 nm is sputtered on the PDMS MNA mold. In order to reduce the deformation of the PDMS due to the downward pressure of the metal during electroplating, the PDMS first-level mold is bonded to the glass to strengthen the tolerance of the PDMS. The electroplating solution was sulfamate (PH = 4), stirring was continued and the temperature was maintained at 37 °C, the current density was 10 mA/cm^2^, and 350 μm thick metal nickel was electroplated at a plating rate of 0.2 μm/min.

### 2.2. Engraving Process

**Figure 2:**
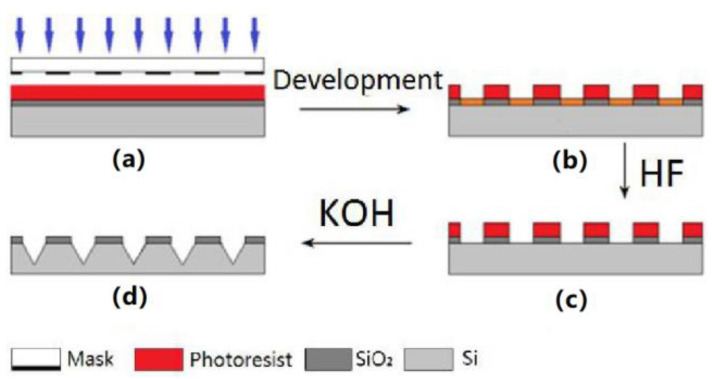
Manufacturing process of MNA tip array. (**a**) Exposure. (**b**) Developing. (**c**) Silica etching. (**d**) Silicon etching.

To achieve low-cost and mass manufacturing of polymer MNA, a MNA mold fabrication method was described. There are two parts of MNA mold: the tip part, which was fabricated by silicon wet etching and the column part, which was fabricated by SU-8 lithography. The fabrication process of the MNA tip is shown in Figure 2. First, we prepared a piece of <100> crystal orientation, a thickness of about 525 μm of double-oxygen single crystal silicon wafer, and washed the surface with oil stain, organic and matter with acetone, alcohol and deionized water which enhanced the bonding force between the silicon wafer and the photoresist. Afterwards, we dried the silicon wafer with pure high-pressure nitrogen gas, and put it into a 180 °C oven for baking for at least 1 h. Secondly, we deposited a layer of AZ4620 series positive photoresist on the oxide layer of the silicon wafer to a thickness of 5 μm, and subjected it to hard bake treatment under the condition of 180 °C for 30 min. Thirdly, photolithography was conducted using CA800 deep ultraviolet lithography machine, for which the exposure intensity was 8.2 mW/cm^2^ and the exposure time was 60 s. As shown in Figure 2a, the mask is a 400 × 400 array of transparent square patterns (200 μm side length), and the structure shown in Figure 2b was fabricated by development. Etching was further conducted in a buffered etchant at 45 °C (solution ratio of 28 mL hydrofluoric acid, 117 g ammonium fluoride and 170 mL deionized water) for 10 min, after the remaining photoresist was removed by ultrasonication with acetone solution. To determine whether the SiO_2_ layer is inscribed, the resistance between the etched holes can be measured with a multimeter, if there is an indication, the etch is successful, as shown in Figure 2c. Finally, the exposed silicon was anisotropically etched at 85 °C with a 30% KOH solution, and the substrate was placed at a 45° in the solution by using a fixture. At the same time, the stirrer was continuously stirred to ensure uniform etching; the etching rate was 1 μm/min, and the time is 2 h. The silicon MNA tip structure is shown in Figure 2d. The silicon wafer was cleaned after the final etching, and the surface profiler can be used for preliminary depth detection to ensure the smooth completion of the experiment.

**Figure 3:**
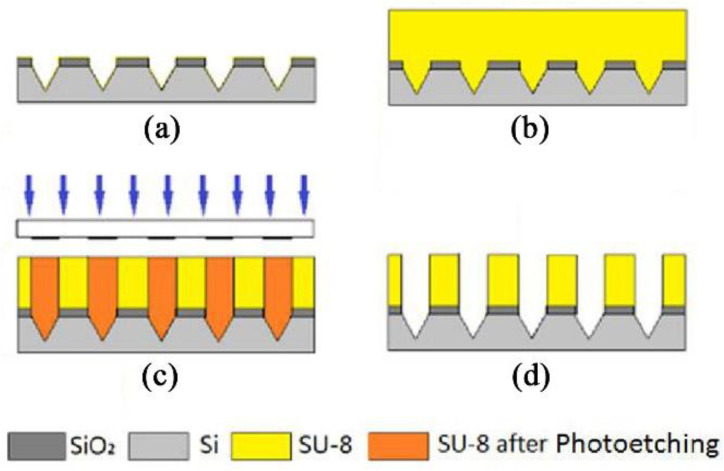
Manufacturing process of the column part of SU-8 MNA. (**a**) Silicon tip. (**b**) Overlay SU-8 photoresist. (**c**) Exposure. (**d**) Developing.

The process of the column part of the MNA is shown in Figure 3. First, a 250 μm thick SU-8 photoresist was deposited on the etched silicon wafer (Figure 3b), which is a negative, epoxy-type and near-ultraviolet photoresist. The exposure of the entire SU-8 photoresist layer is uniform because of low light absorption in the near-ultraviolet range, and a thick film pattern with vertical sidewalls and high aspect ratio can be fabricated. It also has good mechanical properties, chemical resistance, and thermal stability. Photolithography was then performed using an ultraviolet lithography process (Figure 3c), which was an array of 4-hole trench patterns that are developed to provide the structure shown in Figure 3d. This structure is the MNA master mold through which a PDMS MNA can be fabricated. In the experiment, the PDMS pre-polymer was prepared by mixing the base and curing agent at the ratio of 10 and stirred for 5 min, evacuated in a vacuum desiccator to remove air bubbles, and then the PDMS prepolymer was poured onto the master mold with the thickness was about 2 mm, evacuated again in the vacuum desiccator. After that, it was solidified in an oven at 65 °C for 2 h and kept at room temperature to cool down, the PDMS layer was peeled off from the SU-8 master mold and finally a PDMS MNA can be fabricated.

### 2.3. Tilting Rotary Ultraviolet (UV) Lithography

The tilt-rotation exposure process uses a 4-inch photolithography mask. The circular diameter of the mask pattern is 75 μm, the pitch is 300 μm, and the density is 35 × 35 stitches/cm^2^. The mechanism of conical pit formation is shown in the figure below, and the size of the MNA is controllable. The height of the MNA can be changed by adjusting the inclination angle of the bracket and the diameter of the small solid circle on the mask. The formula is as follows:

$$h=\frac{d}{2\tan\theta }$$

where h is the height of the MNA; θ is the tilt angle of the stent; d is the diameter of the small solid circle on the mask.

**Figure 4:**
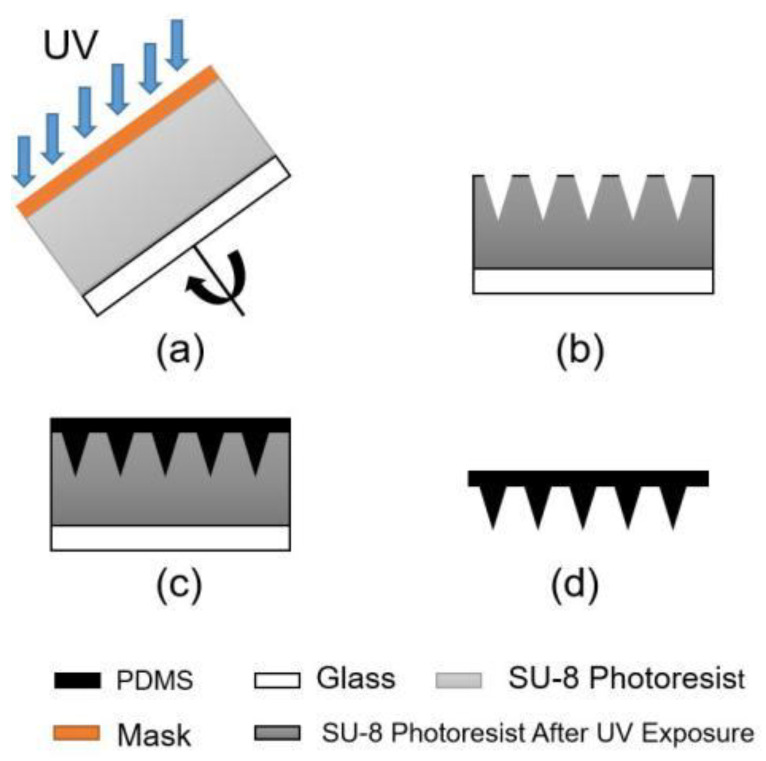
Manufacturing process of Conical MNA. (**a**) Tilting rotary exposure technology. (**b**) Conical array of concavities. (**c**) Polydimethylsiloxane (PDMS) transfer technology. (**d**) PDMS MNA.

**Figure 5:**
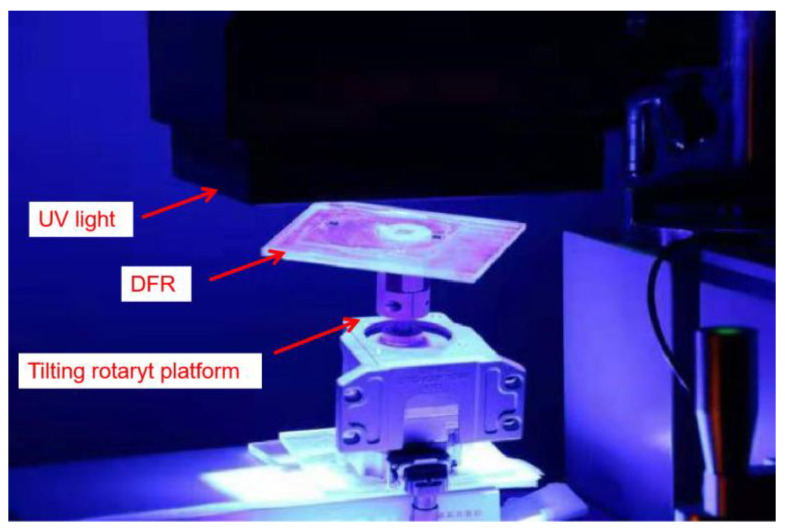
Tilting rotary Ultraviolet (UV) lithography platform.

In this process, a conical array is prepared on the SU-8 using the mask tilting rotary exposure technology on the back side, afterward the PDMS MNA first-level mold is obtained by the PDMS transfer technology. The process flow chart is shown in Figure 4. The inclined and freely rotatable exposure platform consists of a platform and motor which is placed on a freely adjustable bracket. By adjusting the height of the bracket, the rotating platform can be varied from 0° to 90°, as shown in Figure 5. The speed of the motor is controlled applying a DC voltage, so that the motor can drive the rotating platform freely. For exposure, the bench is placed under the URE-2000/35A UV deep lithography mirror without the need to modify the lithography equipment.

In this process, SUEX Dry Film Resist (DFR) replaces the traditional SU-8 photoresist, the thermal lamination process replaces the original silicone technology. DFR is directly adhered to the glass substrate, thereby saving time and efficiency. Moreover, the thickness of the DFR is given, and it is not necessary to adjust the complicated tannin parameters, and the thickness of the DFR used in this experiment is 250 μm. During exposure, the tilt angle of the rotating platform was set to 18°, the platform rotation speed was 400 rpm/min, and the exposure time was 70 s. The lithography mask used in the experiment was a film version with a circular diameter of 75 μm, a center distance of 300 μm, and a density of 35 × 35 needles per square centimeter.

## 3. Results

### 3.1. Fabrication Results of LIGA

#### 3.1.1. Out-Plane Microneedle Array (MNA)

**Figure 6:**
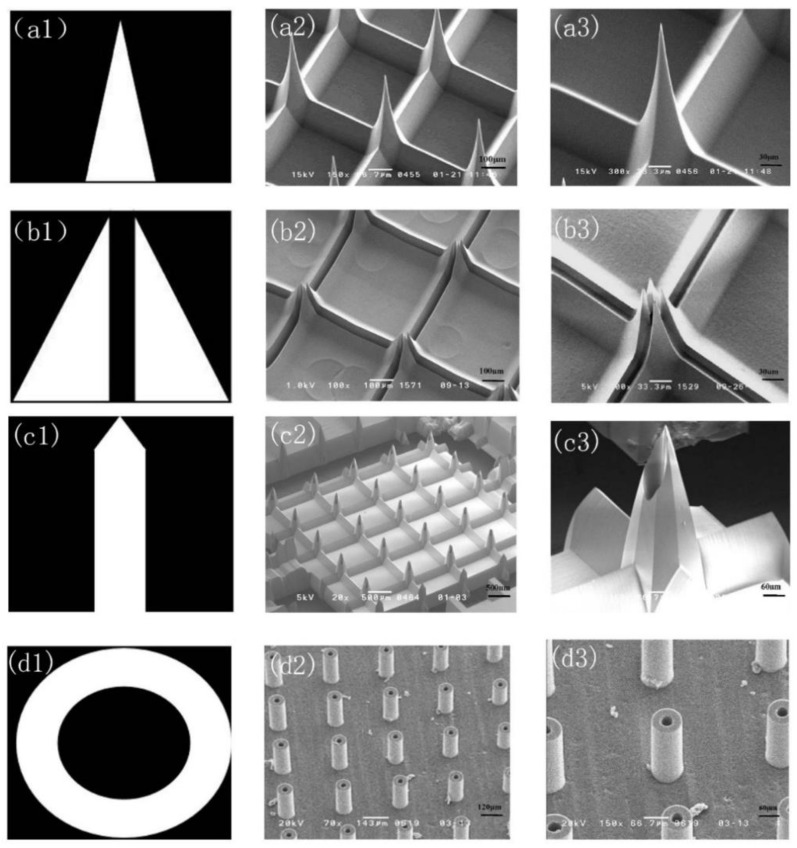
Fabrication results of PMMA out-plane MNA. (**a1**) Triangular absorber pattern. (**a2**) Out-plane solid MNA. (**a3**) An enlarged image of (**a2**). (**b1**) Double right triangle absorber pattern. (**b2**) Out-plane blood extraction MNA. (**b3**) An enlarged image of (**b2**). (**c1**) Obelisk shaped absorber pattern. (**c2**) Out-plane hollow MNA. (**c3**) An enlarged image of (**c2**). (**d1**) Concentric circular absorber pattern. (**d2**) Out-plane hollow MNA. (**d3**) An enlarged image of (**d2**).

By designing the shape of the mask, different styles of microneedle arrays can be prepared. When the mask absorber pattern is a triangle, the PMMA out-plane solid MNA shown in Figure 6(a2) can be obtained by twice moving X-ray exposures, and Figure 6(a3) is a close-up image of a single solid MNA. The MNA height is 350 μm, the bottom width is 100 μm, the center distance of the needle is 330 μm, and the tip width is 2 μm. When the mask pattern of the absorber is as shown in Figure 6(b1), the structure shown in Figure 6(b2) can be fabricated by two moving X-ray lithography, and the density of the PMMA out-plane MNA reaches 1024 needles per square centimeter. This MNA can perform painless micro blood extraction by capillary force. When the obelisk absorber pattern is a pointed shape (Figure 6(c1)), the PMMA out-plane hollow MNA shown in Figure 6(c2) can be manufactured by twice moving X-ray lithography and one alignment fixed exposure. Figure 6(c3) is a close-up image of a single MNA. The MNA have sufficient sharpness to penetrate the skin well, providing a potential for transdermal administration. Figure 6(d2) is an out-plane hollow MNA array obtained by a fixed exposure using a concentric circular mask. The single MNA has a height of 150 μm, an outer diameter of 100 μm, and an inner diameter of 30 μm.

It can be proven by experiments that by using PCT, designing different X-ray mask absorber patterns and different lithography methods can be used to fabricate different plane solid MNA arrays with different shapes and strengths, such as out-plane hollow MNA and in-plane solid MNA.

**Figure 7:**
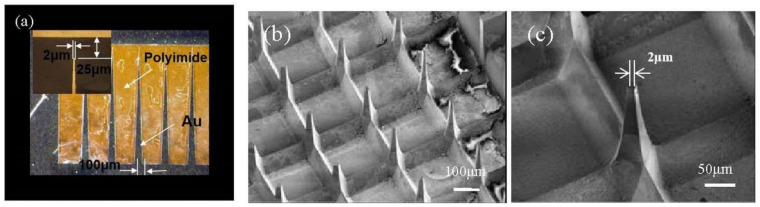
Fabrication results of metal nickel MNA. (**a**) Pattern and size of mask. (**b**) Metal nickel MNA. (**c**) An enlarged image of (**b**).

In the experiment, the mask pattern is an isosceles triangle which the base of the isosceles triangle is 100 μm, the height is 1500 μm, the tip is 2 μm wide, and the tip is 25 μm from the boundary of the mask, as shown in Figure 7a. The gold absorber has a thickness of 3 μm and the polyimide has a thickness of 38 μm. After twice moving X-ray lithography processes (exposure dose of 0.06 Ah), developed in a GG developer at 37 °C for 3 h to obtain a PMMA MNA mold with a height of 300 μm and a tip width of 2 μm. The PMMA solid MNA prepared by the above method is used as a master model, and the secondary PDMS solid MNA is obtained through the transfer process. The secondary MNA is electroplated by the electroplating solution, finally, the metallic nickel MNA is fabricated by electroplating nickel on the PDMS MNA mold. The pattern after electroplating has a good reproduction effect, and the metal MNA has a complete structure and a high aspect ratio. The SEM image as shown in Figure 7b,c.

#### 3.1.2. In-Plane MNA

**Figure 8:**
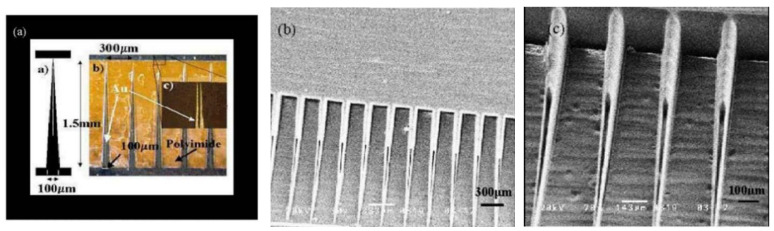
Fabrication results of PMMA in-plane MNA. (**a**) Pattern and size of mask. (**b**) In-plane solid MNA. (**c**) An enlarged image of (**b**).

We introduce a method for manufacturing a PMMA MNA with a tip and a fluid channel, and Figure 8a shows the mask pattern for preparing the MNA. The mask plate used was selected from Au as an absorber which had a thickness of 3 μm, and polyimide as a light-transmitting portion having a thickness of 38 μm. The MNA height and the base width are designed to be 1500 μm and 100 μm, respectively, and the channel width is 10 μm. After a fixed alignment exposure, development on a 0.5 mm PMMA sheet yielded the in-plane MNA array structure shown in Figure 8b,c. The method can obtain a row of MNAs in the same plane, so the preparation of the MNA can be completed by layer-by-layer bonding of 0.5 mm PMMA sheets, and the depth of the pattern can be controlled by the exposure time and the development time. The tip of the prepared MNA is sharp enough to pierce the skin, and the flow channel can be used to store the extracted blood.

### 3.2. Fabrication Results of Engraving Process

**Figure 9:**
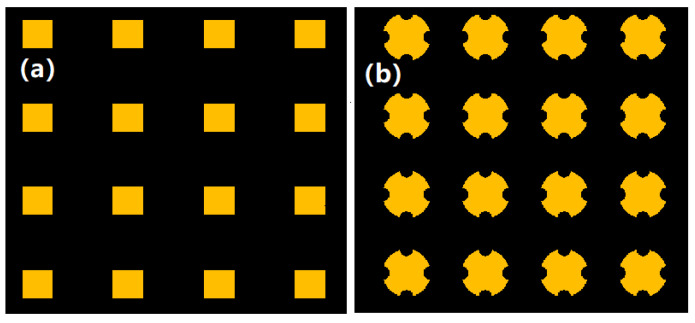
Lithography mask pattern. (**a**) Square pattern. (**b**) Circular pattern with four grooves.

**Figure 10:**
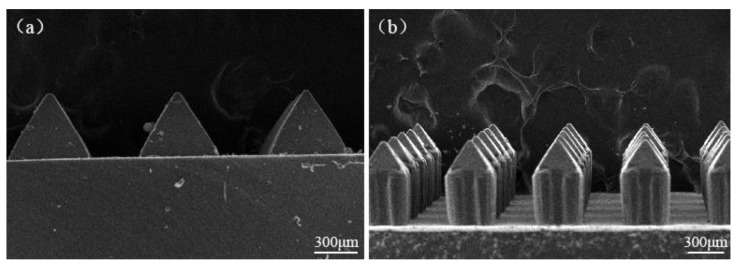
The SEM image of (**a**) MNA tip part and (**b**) the whole MNA.

Figure 9a,b are mask patterns to fabricate the tip part and the column part of MNA, respectively, and the small square in Figure 10a has a side length of 200 μm and a density of 300 needles per square centimeter. The mask pattern in Figure 9b is modified on the basis of Figure 9a. The purpose is to make the prepared MNA column part with four grooves to facilitate demolding and drug loading.

After the original silicon MNA mold is prepared by the silicon wet etching combined with SU-8 lithography, considering that the direct use of the original mold will cause its manufacturing cost to rise, PDMS materials with better biocompatibility and mechanical properties can be cast, and the quadrangular pyramidal MNA can be obtained by using the mold-transforming technique (first-level positive mold). The electron micrograph of the MNA tip and the entire MNA is shown in Figure 10. The overall height of the needle is about 600 μm, the width is about 300 μm, the center distance is about 800 μm, and the density is 300 needles per square centimeter. The tip is anisotropically etched at 54.7°. The results show that the quadrangular pyramid shaped MNAs prepared by this process have a long service life, can be used repeatedly and are resistant to abrasion, can maintain the sharpness of the MNAs, have consistent repeated effects, and meet the requirements of controllable MNA parameters. We then translate the first-level positive mold into the second-level negative mold, and use L-polylactic acid (PLLA), polystyrene (PS), hyaluronic acid (HA) and other polymer materials as the mold transfer material, which can greatly improve the efficiency and success of the transfer technology, and there is no distortion, so as to achieve low-cost, mass production of various polymer MNA arrays.

### 3.3. Fabrication Results of Tilting Rotary UV Lithography

**Figure 11:**
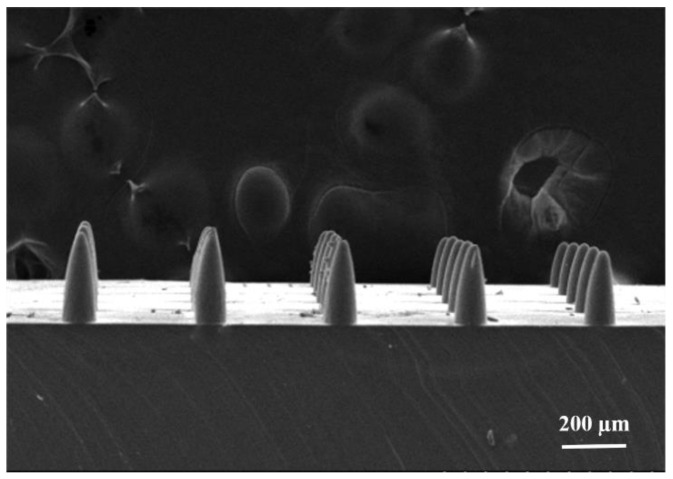
SEM image of conical MNA fabrication result.

After the array of conical pits fabricated by the inclined and rotating UV lithography, a conical MNA was fabricated by PDMS transfer technology. First, we mixed the Dow Corning sygard 184 prepolymer with the curing agent in a ratio of 10:1, vacuum to remove the bubbles, then filled the processed conical MNA mold, vacuum again to remove the bubbles, and solidified it in an oven at 60 °C. After 3 h, the mold was released to obtain a PDMS conical MNA, as shown in Figure 11. According to the SEM image, the actually prepared conical MNA has a width of about 75–80 μm, a height of about 200–210 μm, a taper angle of 5.1–15.6°, a center-to-center distance of about 310-318 μm, and a density of 35 × 35 needles per square centimeter [22]. The results show that the MNA structure with high aspect ratio can be fabricated by this method, and the MNA pattern was copied well and the copy success rate was high. The ultraviolet rotating exposure process was applied to fix the light source, and the mask was rotated by the inclined rotating platform to prepare the conical MNA. At the same time, the hot-pressing process was used to replace the traditional SU-8 photoresist with SUEX Dry Film Resist (DFR), which improves efficiency and convenience. Therefore, the MNA mold can be applied to prepare MNA of various polymer materials.

### 3.4. Comparison of Three Processes and Special Processing Technology

In recent years, special processing technology has developed rapidly. In addition to the above three methods, it also includes some novel preparation methods such as 3D printing technology [23], laser processing [24], drawing lithography [25], etc. The MEMS process can manufacture microneedles with different shapes, angles, and surface contours, and effectively enhance the strength and toughness of the microneedles, enabling them to perform different functions in various fields. The prepared MNA can control the aspect ratio by controlling the exposure time and the development time to meet the requirements of the controllable parameters. The special processing technology does not require a mask, and the use of biodegradable polymer as the material can realize simple and low-cost rapid prototyping, thereby providing mild and rapid preparation conditions. All the above processes can prepare MNA that have a long service life and can be used repeatedly. However, each process has different characteristics, such as the shape and type of MNA, the operability of the preparation process, etc., as shown in Table 1. For the type of MNA, A can prepare in-plane MNA and out-plane MNA, but the other three processes can only prepare out-plane MNA. Regarding the shape of the MNA, B and C can prepare quadrangular pyramid shaped MNA and conical MNA, respectively, but A can prepare MNA of various shapes according to different masks to achieve the purpose of customized production. For D, the layered structure of MNA is prepared using additive manufacturing methods. A and B can prepare solid MNA and hollow MNA, but B and C can only prepare solid MNA by wet etching of silicon combined with SU-8 photolithography and tilting rotary exposure. The aspect ratio of A is greater than 2, up to 30. However, the equipment used in this process is expensive, the process is complicated, and the process requirements are high. In terms of B, C, and D, the aspect ratio of about 5 can be achieved through lower cost and simple process flow. Regarding the processing methods, A, B, and C are manufactured in parallel, which saves time and has good reproducibility, while D is manufactured in serial, which wastes time and has a larger error range. Regarding A, its preparation cost is high, but its accuracy and scalability can reach the nano-level. However, for B, C, and D, due to the lower cost, the accuracy and scalability of the prepared MNA can only be reach the micron-level. With respect to B, the tip is prepared by an anisotropic etching method, so the etching is performed at an angle of 54.7°. The cutting-edge precision prepared by C and D can reach the μm level, while the A can reach the nano-level.

## 4. Conclusions

This paper introduces three methods for manufacturing MNA based on MEMS technology. The first method is to obtain the PMMA MNA based on the PCT process, which is prepared by different exposure methods and masks of different shapes, and then the metal nickel MNA mold can be prepared by the nickel-plating process. The MNA prepared by this method can be used for blood extraction and drug injection. The second method is to use the wet etching of silicon and UV-LIGA process to prepare the MNA tip part and the SU-8 photoresist column part respectively, which constitutes the MNA mold as a whole, and the PDMS transfer technology can be used to different out-plane MNA structure. The last method is to prepare a conical MNA mold on a SU-8 dry film by using inclined and rotating UV lithography, and finally, a conical MNA is obtained by a transfer process.

The experimental results indicate that these three methods can fabricate MNA with high aspect ratios and sharp tips, which provides a good guarantee for the MNA to penetrate the skin. We discovered that the error range of reproducibility and accuracy is 2–11% by compared the dimensions of designed and actual MNs. For the PCT technology, although the cost is high and the process is complicated, of in-plane and out-plane can be prepared for blood extraction and drug injection, due to the hollow MNA having a very high aspect ratio. For the engraving process and the tilting rotary exposure process, they are simple to operate, and the prepared MNA mold has good repeatability and accuracy. After that, PDMS MNA molds can be obtained by employing the PDMS transfer technology. Using these molds, it is possible to prepare drug loaded MNA of dissolved materials, which further provides support for research on transdermal drug delivery. Through comparison with special processing technology, we found that MNA prepared based on MEMS technology have many characteristics such as parallel manufacturing, good reproducibility, high processing accuracy, time saving, etc. The MEMS process can manufacture MNA with different shapes, angles, and surface contours, and effectively enhance the strength and toughness of the microneedles, enabling them to perform different functions in various fields.

## Figures and Tables

**Table 1:** Comparison of Three Processes and Special Processing Technology.

Process Comparison Items	LIGA (A)	Engraving Process (B)	Tilting Rotary UV Lithography (C)	Special Processing Technology (D)
Type of MNA	in-plane MNA and out-plane MNA	out-plane MNA	out-plane MNA	out-plane MNA
Shape of MNA	The shape of the MNA changes according to the shape of the mask	quadrangular pyramid shaped MNAs	Conical MNA	Layered MNA
Hollow MNA	Yes	No	No	Yes
Experimental steps	Subtractive manufacturing	Subtractive manufacturing	Subtractive manufacturing	Additive manufacturing
aspect ratios	Up to 30:1	~2:1	~2:1	Up to 5:1
Fabrication costs	expensive	Cheap	cheap	cheap
Fabrication time	save time	save time	save time	Time consuming
Material	PMMA	Si + SU-8	SU-8	Resin/polymer
Geometrical capability	Cutting-edge accuracy reaches the nano-level	the tip is anisotropically etched at 54.7°	Cutting-edge accuracy reaches micron-level	Cutting-edge accuracy reaches micron-level
Accuracy	Up to 0.1 μm	Up to 10 μm	Up to 10 μm	Up to 10 μm
Reproducibility	coincident	Coincident	coincident	erroneous
Scalability	nm-μm	Μm	μm	μm
Processing methods	Parallel manufacturing	Parallel manufacturing	Parallel manufacturing	Serial manufacturing

## Data Availability

Data available on request due to restrictions e.g., privacy or ethical. The data presented in this study are available on request from the corresponding author.
